# Crohn’s and Colitis Canada’s 2021 Impact of COVID-19 and Inflammatory Bowel Disease in Canada: Risk Factors and Medications

**DOI:** 10.1093/jcag/gwab032

**Published:** 2021-11-05

**Authors:** Laura E Targownik, Charles N Bernstein, Peter L Lakatos, Sanjay K Murthy, Eric I Benchimol, Alain Bitton, James Guoxian Huang, M Ellen Kuenzig, Jennifer L Jones, Gilaad G Kaplan, Kate Lee, Mariam S Mukhtar, Parul Tandon, Joseph W Windsor, Remo Panaccione

**Affiliations:** 1 Division of Gastroenterology and Hepatology, Mount Sinai Hospital, University of Toronto, Toronto, Ontario, Canada; 2 Department of Internal Medicine, Max Rady College of Medicine, Rady Faculty of Health Sciences, University of Manitoba, Winnipeg, Manitoba, Canada; 3 University of Manitoba IBD Clinical and Research Centre, Winnipeg, Manitoba, Canada; 4 Department of Medicine, McGill University Health Centre, McGill University, Montreal, Quebec, Canada; 5 First Department of Medicine, Semmelweis University, Budapest, Hungary; 6 The Ottawa Hospital IBD Centre, Department of Medicine, University of Ottawa, Ottawa, Ontario, Canada; 7 SickKids Inflammatory Bowel Disease Centre, Division of Gastroenterology, Hepatology and Nutrition, The Hospital for Sick Children, Toronto, Ontario, Canada; 8 Child Health Evaluative Sciences, SickKids Research Institute, Toronto, Ontario, Canada; 9 ICES, Toronto, Ontario, Canada; 10 Department of Paediatrics and Institute of Health Policy, Management and Evaluation, University of Toronto, Toronto, Ontario, Canada; 11 Department of Medicine, Dalhousie University, Halifax, Nova Scotia, Canada; 12 Department of Medicine, University of Calgary, Calgary, Alberta, Canada; 13 Department of Community Health Sciences, University of Calgary, Calgary, Alberta, Canada; 14 Crohn’s and Colitis Canada, Toronto, Ontario, Canada; 15 Department of Internal Medicine, King Abdulaziz University Hospital, Jeddah, Saudi Arabia; 16 Division of Gastroenterology and Hepatology, Department of Medicine, University of Calgary, Calgary, Alberta, Canada

**Keywords:** Biologics, COVID-19, Inflammatory bowel disease, Risk factors

## Abstract

Inflammatory bowel disease (IBD) is a disease that results from dysregulation of the immune system and frequently requires medications that can affect the immune response to infections; therefore, it was imperative to quickly understand the risk of coronavirus disease 2019 (COVID-19) infection on persons living with IBD and how that risk may be increased by commonly used IBD medications. The IBD research community in Canada and beyond quickly established collaborative efforts to better understand the specific risk posed by COVID-19 on persons with IBD. We learned that IBD itself was not a risk factor for death or serious complications of COVID-19, and that most commonly used drug classes (with the notable exception of corticosteroids) do not increase the risk of COVID-19-related adverse outcomes. The risk factors for serious complications and death from COVID-19 appear to be similar to those identified in the wider population; those being advanced age, having pre-existing heart or lung disease, and smoking. We recommend that persons with IBD do not alter their course of therapy to avoid complications of COVID-19, though the indiscriminate use of corticosteroids should be avoided. Persons with IBD should follow the same public health recommendations as the general population to reduce their personal risk of acquiring COVID-19.

**Key Messages**

- Most people with IBD are not at increased risk of severe COVID-19 or death from COVID-19 as compared to the general population.
- No biologic medication has been shown to increase the risk of severe COVID-19 or death from COVID-19.
- Persons with IBD who are flaring and require a high dose of corticosteroids are at higher risk of acquiring or experiencing severe complications to COVID-19.
- Persons with IBD should stay on therapy to maintain remission as discontinuation of treatment due to fear of COVID-19 may lead to a flare requiring corticosteroids, which elevates the risk of worse outcome for COVID-19.

## Introduction

At the outset of the pandemic, it was paramount to identify factors associated with an increased risk of contracting severe acute respiratory syndrome coronavirus 2 (SARS-CoV-2) and, more importantly, which factors were associated with poor outcomes such as the need for hospitalization, ICU admission, mechanical ventilation or death. This concern was particularly acute among persons living with chronic immune-mediated diseases like inflammatory bowel disease (IBD), who not only suffer from immune dysfunction but are often on therapies that may affect their systemic immune system (1). At the beginning of the pandemic, it was unknown if persons with IBD would be more susceptible to infection and, if infected, at increased risk of poor outcomes.

Persons with IBD are more likely to suffer from infectious diseases than the general population (2). Prior to the emergence of coronavirus disease 2019 (COVID-19), several factors had already been identified that were associated with an increased risk of infections in people with IBD, including underlying disease activity, malnutrition, advanced age and certain therapies used to treat IBD. Many of these medications, in particular corticosteroids, purine anti-metabolites and anti-tumour necrosis factor (anti-TNF) biologics, have been associated with an increased risk of serious and opportunistic infections (3–7). Hence, there was concern that these may also be risk factors for contracting SARS-CoV-2 and developing severe complications of COVID-19. The most important questions to answer for persons living with IBD were:

1. Which individuals with IBD are at increased risk of poor outcomes of COVID-19?
2. Which medications commonly used by people with IBD increase the risk of poor outcomes from COVID-19?
3. What are the steps that individuals living with IBD who are at higher risk for serious complications of COVID-19 can take to reduce their personal risk?

Over the past year, there has been a significant expansion in our knowledge and many key learnings, including in the field of IBD. During this time, recommendations were updated in response to updated knowledge, especially about the behaviour of the virus and strategies that were most effective in limiting its spread and its impact. In this article, we summarize our understanding about the risk of serious COVID-19 faced by persons with IBD, recommendations made by the Crohn’s and Colitis Canada COVID-19 and IBD Taskforce to Canadians living with IBD and their health care providers regarding COVID-19 risk management, and what questions still remain unanswered.

## GATHERING INFORMATION

The first article to specifically address how the novel coronavirus infection might impact persons with IBD reported that, among the 20,000 persons with IBD followed at China’s seven largest IBD centres, none had yet been diagnosed with COVID-19 (8); this was the same as the early reports emerging out of Italy (9). By this time, advanced age, chronic cardiorespiratory disease, malignancy and obesity were being recognized as major contributors to COVID-19-related morbidity and mortality (10). Moreover, there did not appear to be an obvious signal of more severe outcomes in persons who either have diseases that suppress the immune system, or who are treated with immunosuppressive medications. However, as the risk of serious infection with COVID-19 had not yet been well characterized in persons with IBD, it was unclear whether persons with IBD should practise greater physical distancing and other preventative measures than persons who were otherwise healthy.

**Table 1.** Risk factors for worsened COVID-19 outcomes based on IBD medication

Drug class	Direction of increased risk
5-ASAs	0
Corticosteroids	
Over 20 mg/day in prednisone equivalents	++
Less than 20 mg/day in prednisone equivalents	?
Budesonide	0
Immunomodulators	+
Thiopurines (azathioprine/6-MP)	+
Methotrexate	+
Anti-TNFs	0
As monotherapy	0
In combination with thiopurines/methotrexate	+
Vedolizumab	0
Ustekinumab	0
Tofacitinib	0

0: no increased risk or no evidence of increased risk; ?: uncertain risk; +: possible increased risk or probable small increased risk (use with caution in settings where risk of COVID-19 acquisition is appreciable); ++: definite increased risk (avoid unless no alternatives in settings where risk of COVID-19 acquisition is appreciable). ASA, Aminosalicylate acid; IBD, Inflammatory bowel disease; TNF, Tumour necrosis factor; 6-MP, 6-mercaptopurine.

To understand the true impact of COVID-19 on the IBD community would require a large and comprehensive database of individuals with IBD who were diagnosed with COVID-19. The IBD research community was quick to mobilize to gather data on the IBD-specific risk of COVID-19 and established the Surveillance Epidemiology of Coronavirus Under Research Exclusion (SECURE-IBD) registry (11). This initiative sought physicians to register confirmed cases of COVID-19 among persons with IBD from all over the world. Within 1 month, there were sufficient data to draw some preliminary impressions about which individual and disease-related characteristics were potentially associated with an increased risk of serious COVID-19, defined as infections requiring hospitalization, ICU admission and/or mechanical ventilation, or those resulting in death. As more cases were reported to this registry, our understanding of the risk of a severe COVID-19 became more apparent. Despite, the uncertainty of the representativeness of the cases included in the SECURE-IBD registry, these data provide further reassurance as to the mild course of COVID-19 in the vast majority of people with IBD without additional non-IBD-related risk factors (12). Studies on risk factors for severe COVID-19 in those with IBD have also been corroborated in other study populations outside of the SECURE-IBD registry (13).

As of the end of April 2021, the SECURE-IBD database has nearly 6000 cases, of whom 15% were hospitalized for severe COVID-19 (14). The overall rate of requiring ICU admission or dying from COVID-19 was 4%. The strongest predictors of more serious COVID-19 or dying was advanced age (12% if aged 60 to 69 years, 18% if aged 70 to 79 and 25% if aged over 80 years), and having multiple other chronic medical conditions (33% rate of ICU admission or death in those with three or more chronic medical conditions, including IBD). For people under the age of 50, the risk of ICU admission or dying was around 1%. However, individuals under 50 with more severe disease activity were more likely to require hospitalization and have severe COVID-19 outcomes compared to those with mild disease or those in remission (15). Aside from corticosteroid use (13% risk of ICU admission or death), the risk of these more serious outcomes was low, ranging from 1% for persons using anti-TNF monotherapy to 7% for persons using azathioprine or methotrexate. These results were not adjusted for age, so once again it could be that the higher rates of serious disease in persons using these drugs may be related to other factors, like the age and overall non-IBD health of those persons.

**Figure 1.**
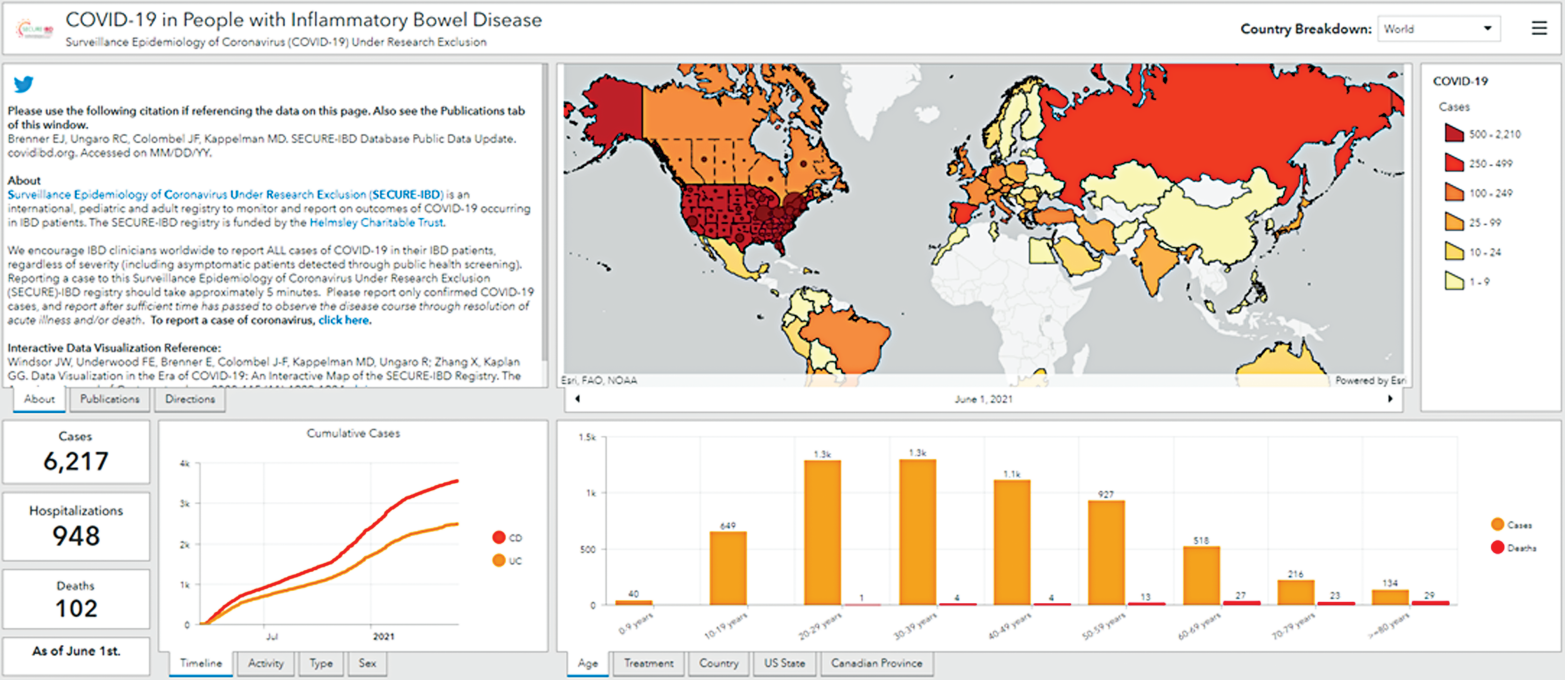
Interactive online dashboard and map of COVID-19 cases in those with inflammatory bowel disease (IBD) derived from the Surveillance Epidemiology of Coronavirus Under Research Exclusion (SECURE-IBD) database on June 1, 2021: Link to online interactive map: https://kaplan-secure-ibd-ucalgary.hub.arcgis.com/. Persons at the highest risk were those who were of 65 years of age or older, those with serious non-IBD-related medical comorbidities like advanced cardiovascular and pulmonary disease, those using over 20 mg of prednisone daily, those with moderate-to-severe inflammation or malnutrition and those on total parenteral nutrition. Persons at moderate risk were those under the age of 65 who were using immunomodulatory or biologic. All other persons with IBD were classified as being at average risk (i.e., the same as the general population).

A research team at the University of Calgary developed an interactive online dashboard to visualize data from the SECURE-IBD registry such as depicting the IBD cases with COVID-19 by time, country, age, sex, disease type, disease activity and medication usage (Figure 1), which serves as a useful resource to the IBD community for accessing up-to-the-moment knowledge on COVID-19 risk factors for IBD (16). Additionally, the SECURE-IBD consortium has also developed an interactive tool to allow users to calculate their risk of developing severe COVID-19, based on the demographic and disease-related risk factors, which can be used to guide decisions about risk avoidance. The association between severe COVID-19 and the medications used to treat IBD are outlined below with recommendations in a subsequent section.

## MEDICATIONS, DISEASE ACTIVITY AND OTHER RISK FACTORS

A summary of the association of commonly used IBD drugs and the risk of COVID is shown in Table 1.

### Steroids

The use of corticosteroids, particularly at doses above 20 mg daily, was more frequently seen among persons who developed severe COVID-19. This may reflect the high levels of systemic immunosuppression resulting from high-dose steroid use and/or the severity of active disease.

### Anti-TNF Therapy

In the initial release of data, there was no definite signal that anti-TNF use was associated with a higher risk of severe COVID-19.

### 5-Aminosalicylate Acid

Although the initial analysis found an association between 5-aminosalicylate acid (5-ASA) use and a higher rate of adverse outcomes, this was not believed by most to be plausible, given that 5-ASAs are not known to have a systemic immunosuppressive effect. In subsequent analyses on a larger number of individuals, the association between 5-ASAs and adverse events was no longer detected.

### Immunomodulators

The most recent publication from SECURE-IBD in October 2020 detailed COVID-19 infections in 1439 persons with IBD in 47 countries. The main finding regarding medications was that the use of thiopurines, with or without anti-TNF therapy, was associated with a fourfold higher risk of developing severe COVID-19 outcome (hospitalization and/or death) than using anti-TNF therapy alone (14).

### Newer Biologics: Vedolizumab and Ustekinumab

Persons who were using vedolizumab were not found to be at higher risk of severe COVID-19 outcomes when compared to people using anti-TNFs (17). There are no definitive data on the risk associated with ustekinumab, though this agent, like vedolizumab, has not been associated with an increased risk of infection in general (18). Furthermore, ustekinumab use has not been shown to increase the risk of COVID-19 in persons with psoriasis, another autoimmune condition where ustekinumab is commonly used (19).

### Small Molecules: Tofacitinib

By December 2020, the SECURE-IBD group also reported that users of tofacitinib were not at increased risk of severe COVID-19 when compared to users of biologic medications (20).

### Disease Activity

Early publications from the SECURE-IBD registry described a higher risk of severe COVID-19 among individuals with severely active IBD. Further analyses of this association indicated that moderately and severely active IBD (as defined by the Physician’s Global Assessment) is associated with hospitalization; severely active IBD was associated with ICU admission, mechanical ventilation or death only in people ≤50 years of age, after adjusting for corticosteroid use (15).

### Other Risk Factors

Throughout the publications from the SECURE-IBD registry, rates of severe COVID-19 were highest in persons with advanced age, and those with multiple concomitant medical comorbidities; this is similar to the general population.

Importantly, data from population-based European health care registries have also suggested that having IBD on its own likely did not increase the risk of COVID-19 (21–23). Therefore, independent of medication use, the risk of more serious COVID-19 in persons with IBD was due mostly from the same types of risk factors that are seen in the general population, including advancing age, chronic lung and heart disease, obesity and smoking. At this time, there is no definite evidence that having previously undergone intestinal surgery increases the risk of developing more serious COVID-19. However, inflammation and a history of intestinal resections can increase the risk of malnutrition, and malnutrition may be a risk factor for more severe COVID-19. Therefore, the risk of more severe outcomes for people with more active IBD must still be considered.

## TASKFORCE RECOMMENDATIONS

Based on the initial data from SECURE-IBD as well as applying what is known generally about the risk factors for infection in IBD, the Crohn’s and Colitis Canada COVID-19 and IBD Taskforce developed its first set of risk-based recommendations on how to limit the acquisition of SARS-CoV-2 and the development of COVID-19. These recommendations are updated regularly as new information became available about the impact of IBD therapies on the risk of severe COVID-19. Persons with IBD were separated into three risk categories, with different levels of physical distancing and shielding recommended based on the degree of risk. These recommendations were based both on data from SECURE-IBD and extrapolated from factors known to increase the risk of infection-related morbidity in the general population.

The Taskforce also stressed the importance of not abruptly discontinuing effective medications without a discussion of the risks and benefits with their IBD specialists and/or primary care providers. Also, although newer biologic agents such as vedolizumab and ustekinumab are thought to have a lower risk of infections, it is unknown about their impact in the setting of COVID-19. In addition, there were some data that even suggested that persons using anti-TNF agents may be at lower risk than non-users for developing severe COVID-19. However, it is premature to consider anti-TNF users to be at lower risk than other persons with IBD. The Taskforce also advised physicians to avoid prescribing corticosteroids unless there were no other reasonable alternatives, given the higher rate of COVID-19 seen in the preliminary SECURE-IBD data along with the known risks posed by corticosteroid use for infection.

Overall, while the emergence of COVID-19 has increased the level of concern among persons with IBD and the people involved in their care, the overall impact of IBD on COVID-19-related adverse outcomes would appear to be fairly small. The risk of severe COVID-19 outcomes is primarily driven by non-IBD-related factors, like heart and lung health, age and being excessively over or underweight. Corticosteroid use appears to significantly increase the risk of severe COVID-19, and the indiscriminate use of corticosteroids should be discouraged. As for other medications, their impact on COVID-19 risk is likely small. The Taskforce recommends that any decisions made about medications in persons with COVID-19 should be made under the close monitoring of a gastroenterologist.

We also recommend that persons with IBD continue to follow public health advice on physical distancing, mask wearing, being promptly tested for symptoms suggestive of COVID-19 or if exposed to a known case of COVID-19 and be vaccinated at the first available opportunity. It is hoped that over the coming year, with greater rates of vaccination, that the risk of acquiring COVID-19 will be significantly reduced, and with it, the concern about the impact it has on IBD.

## References

[1] Rubin DT , Abreu MT, Rai V, et al.; International Organization for the Study of Inflammatory Bowel Disease. Management of patients with Crohn’s disease and ulcerative colitis during the coronavirus disease-2019 pandemic: Results of an international meeting. Gastroenterology 2020;159(1):6–13.e6. 32272113 10.1053/j.gastro.2020.04.002 PMC7194599

[2] Irving PM , de Lusignan S, Tang D, Nijher M, Barrett K. Risk of common infections in people with inflammatory bowel disease in primary care: A population-based cohort study. BMJ Open Gastroenterol 2021;8(1):e000573. 10.1136/bmjgast-2020-000573 PMC7893652 33597152

[3] Kirchgesner J , Lemaitre M, Carrat F, et al. Risk of serious and opportunistic infections associated with treatment of inflammatory bowel diseases. Gastroenterology 2018;155(2):337–46.e10. 29655835 10.1053/j.gastro.2018.04.012

[4] Brassard P , Bitton A, Suissa A, et al. Oral corticosteroids and the risk of serious infections in patients with elderly-onset inflammatory bowel diseases. Am J Gastroenterol 2014;109(11):1795–802; quiz 1803. 25267328 10.1038/ajg.2014.313

[5] Toruner M , Loftus EV Jr, Harmsen WS, et al. Risk factors for opportunistic infections in patients with inflammatory bowel disease. Gastroenterology 2008;134(4):929–36. 18294633 10.1053/j.gastro.2008.01.012

[6] Osterman MT , Haynes K, Delzell E, et al. Effectiveness and safety of immunomodulators with anti-tumor necrosis factor therapy in Crohn’s disease. Clin Gastroenterol Hepatol 2015;13(7):1293–301.e5; quiz e70, e72. 25724699 10.1016/j.cgh.2015.02.017 PMC4475667

[7] Lichtenstein GR , Feagan BG, Cohen RD, et al. Serious infection and mortality in patients with Crohn’s disease: More than 5 years of follow-up in the TREAT registry. Am J Gastroenterol 2012;107(9):1409–22. 22890223 10.1038/ajg.2012.218 PMC3438468

[8] Mao R , Liang J, Shen J, et al.; Chinese Society of IBD, Chinese Elite IBD Union; Chinese IBD Quality Care Evaluation Center Committee. Implications of COVID-19 for patients with pre-existing digestive diseases. Lancet Gastroenterol Hepatol 2020;5(5):425–7. 32171057 10.1016/S2468-1253(20)30076-5 PMC7103943

[9] Norsa L , Indriolo A, Sansotta N, et al. Uneventful course in patients with inflammatory bowel disease during the severe acute respiratory syndrome coronavirus 2 outbreak in Northern Italy. Gastroenterology 2020;159(1):371–2. 32247695 10.1053/j.gastro.2020.03.062 PMC7270273

[10] Williamson EJ , Walker AJ, Bhaskaran K, et al. Factors associated with COVID-19-related death using OpenSAFELY. Nature 2020;584(7821):430–6. 32640463 10.1038/s41586-020-2521-4 PMC7611074

[11] Surveillance Epidemiology of Coronavirus Under Research Exclusion (SECURE-IBD). <www.covidibd.org> (Accessed March 30,2021).

[12] Brenner EJ , Ungaro RC, Gearry RB, et al. Corticosteroids, but not TNF antagonists, are associated with adverse COVID-19 outcomes in patients with inflammatory bowel diseases: Results from an international registry. Gastroenterology 2020;159(2):481–91.e3. 32425234 10.1053/j.gastro.2020.05.032 PMC7233252

[13] Khan N , Patel D, Xie D, et al. Impact of anti-tumor necrosis factor and thiopurine medications on the development of COVID-19 in patients with inflammatory bowel disease: A nationwide veterans administration cohort study. Gastroenterology 2020;159(4):1545–6.e1. 32479823 10.1053/j.gastro.2020.05.065 PMC7258834

[14] Ungaro RC , Brenner EJ, Gearry RB, et al. Effect of IBD medications on COVID-19 outcomes: Results from an international registry. Gut 2021;70(4):725–32. 33082265 10.1136/gutjnl-2020-322539 PMC8136807

[15] Ricciuto A , Lamb C, Kuenzig E, et al. Disease activity is associated with Covid-19 outcomes in IBD patients with effect modification by age. Gastroenterology 2021;160(6):S330–11.

[16] Windsor JW , Underwood FE, Brenner E, et al. Data visualization in the era of COVID-19: An interactive map of the SECURE-IBD registry. Am J Gastroenterol 2020;115(11):1923–4. 33156119 10.14309/ajg.0000000000000953

[17] Agrawal M , Zhang X, Brenner EJ, Ungaro RC, Kappelman MD, Colombel JF. The impact of vedolizumab on COVID-19 outcomes among adult IBD patients in the SECURE-IBD registry. J Crohns Colitis 2021. doi:10.1093/ecco-jcc/jjab071. PMC8083188 33884425

[18] Engel T , Yung DE, Ma C, et al. Effectiveness and safety of ustekinumab for Crohn’s disease; systematic review and pooled analysis of real-world evidence. Dig Liver Dis 2019;51(9):1232–40. 31202609 10.1016/j.dld.2019.05.002

[19] Kridin K , Schonmann Y, Damiani G, et al. Tumor necrosis factor inhibitors are associated with a decreased risk of COVID-19-associated hospitalization in patients with psoriasis-a population-based cohort study. Dermatol Ther 2021;34(4):e15003. doi:10.1111/dth.15003. 34033207 PMC8209905

[20] Agrawal M , Brenner EJ, Zhang X, et al. Characteristics and outcomes of IBD patients with COVID-19 on tofacitinib therapy in the SECURE-IBD registry. Inflamm Bowel Dis 2021;27(4):585–9. 33325523 10.1093/ibd/izaa303 PMC7799122

[21] Attauabi M , Poulsen A, Theede K, et al. Prevalence and outcomes of COVID-19 among patients with inflammatory bowel disease—a Danish prospective population-based cohort study. J Crohns Colitis 2021;15(4):540–50. 33035299 10.1093/ecco-jcc/jjaa205 PMC7797764

[22] Ludvigsson JF , Axelrad J, Halfvarson J, et al. Inflammatory bowel disease and risk of severe COVID-19: A nationwide population-based cohort study in Sweden. United European Gastroenterol J 2021;9(2):177–92. 10.1002/ueg2.12049 PMC8014882 33704918

[23] Singh AK , Jena A, Kumar-M P, et al. Risk and outcomes of coronavirus disease in patients with inflammatory bowel disease: A systematic review and meta-analysis. United European Gastroenterol J 2021;9(2):159–76. 10.1177/2050640620972602 PMC8250629 33210980

